# Leaving on a jet plane: the trade in fraudulently obtained airline tickets

**DOI:** 10.1007/s10611-018-9777-8

**Published:** 2018-05-08

**Authors:** Alice Hutchings

**Affiliations:** Department of Computer Science & Technology, University of Cambridge, William Gates Building, Cambridge, CB3 0FD UK

## Abstract

Every day, hundreds of people fly on airline tickets that have been obtained fraudulently. This crime script analysis provides an overview of the trade in these tickets, drawing on interviews with industry and law enforcement, and an analysis of an online blackmarket. Tickets are purchased by complicit travellers or resellers from the online blackmarket. Victim travellers obtain tickets from fake travel agencies or malicious insiders. Compromised credit cards used to be the main method to purchase tickets illegitimately. However, as fraud detection systems improved, offenders displaced to other methods, including compromised loyalty point accounts, phishing, and compromised business accounts. In addition to complicit and victim travellers, fraudulently obtained tickets are used for transporting mules, and for trafficking and smuggling. This research details current prevention approaches, and identifies additional interventions, aimed at the act, the actor, and the marketplace.

## Travel fraud—An introduction

Travel fraud, for the purpose of this research, refers to the sale and use of airline tickets that have been obtained illicitly. These tickets are often obtained using stolen credit cards and online credentials. While evidence for this can be seen on online blackmarkets, the nature of the problem, and how this relates to other crimes, has been rather unclear for some time. Different industry segments see differing aspects of the problem, and understanding the nature of this fraud type in its entirety can be challenging. This research addresses this gap by examining advertisements on online blackmarkets, including feedback from unsatisfied customers, and information from law enforcement and industry obtained through interviews with relevant stakeholders. The relationship between those travelling on the tickets, and those obtaining them fraudulently, if they are not the same people, is examined. Using crime script analysis, the research provides an understanding of how this crime works and what countermeasures can be implemented in order to make it far more difficult and less lucrative for the criminals.

Online blackmarkets are used to trade in goods, including drugs and drug paraphernalia, credit card details, electronics, weapons, and services, including those that enable fraudulent activities [1–6]. Some blackmarkets are hosted as hidden services on anonymous networks such as Tor.^1^ Visitors to these sites may find vendors operating as travel agents, organising plane tickets, hotel accommodation, and car rentals, advertised at around 20–75% of market price. The belief that the vendors were involved in some level of criminal activity when it came to making these arrangements spurred this line of enquiry. It was hypothesised the methods to obtain tickets included using stolen payment information, or having insiders within the travel industry (e.g. travel agents, airline staff) make the bookings.

Credit card data can be compromised in a variety of ways, including data breaches, skimmers, malware, phishing, or through employees working in the financial, retail or service industries [6, 9, 10]. To be of worth, stolen credit cards must be monetised by those that hold them. Airline tickets, at first sight, appear to be an excellent way of doing this as they are of high value, and are delivered electronically. Another possibility is the vendors are ‘rippers’,^2^ with unwitting purchasers sending their money, and perhaps their passport and other identification details, and receiving nothing in return.

Some policing currently takes place in relation to travel fraud. Most notably, Europol runs ‘Global Airline Action Days’ on a regular basis, to identify and detain those travelling on tickets purchased using stolen credit card details. These operations, which are run in cooperation with the airline, travel, and payment card industries, have resulted in 2090 suspicious transactions being identified, and 1078 people being detained since 2013 [12–19]. Europol allege those arrested have been travelling in order to commit other offences, including ‘trafficking of human beings, illegal immigration, smuggling of goods, drug trafficking, fraud, cybercrime and terrorism’ [15]. While it is unknown where the tickets were traded, the increasing role of ‘fake online travel agencies’ has been identified [16]. In 2015, Europol estimated the loss to the airline industry as a result of fraudulent ticket purchases to be €1Bn [15], although the various methodological issues when it comes to estimating the extent and impact of online fraud is noted [10]. Difficulties with detecting and reporting travel fraud contributes to the ‘dark figure’ of crime, making quantification particularly difficult [20].

There is evidence of other methods being used to obtain tickets, in addition to compromised card details. In the US in June 2017, a man was sentenced to over four years in federal prison after pleading guilty to wire fraud. He used phishing techniques to obtain credentials for Global Distribution System (GDS) companies, which provide booking services to travel agents. These credentials were used to issue fraudulent airline tickets valued at over US$2 m, which were sold or used personally by him and his accomplices. The tickets were mainly sold in West Africa, and he had been extradited to the US from France, after travelling there from Morocco using a fraudulent passport [21]. There are other indicators of the close link between travel fraud and other types of crime. For example, two people prosecuted in the UK for phishing were purchasing air tickets to allow foreign national offenders to commit crime on London’s transport network [22].

Travel fraud often crosses borders, which makes it particularly difficult to detect and police. For international flights, this involves physically travelling from one jurisdiction to another. However, it can also involve airlines, travel agencies, financial institutions, and individual account holders in multiple countries [23]. This can create difficulties for law enforcement when it comes to navigating the differing criminal justice systems in which the offenders operate [20, 24]. These global aspects create opportunities for offenders, who can target victims’ credit card details in one country and use them to book flights with an airline in another country, while travelling to and from completely different jurisdictions.

As noted by Payne [25], interdisciplinary efforts can expand and enhance criminological research, particularly relating to cybercrime. In computer science, the travel industry has been of interest to computer security researchers for some time. For example, over 10 years ago it was demonstrated that some frequent flyer accounts were accessible if you knew the account number, which at the time was printed on boarding passes [26]. As technology has advanced, so have the vulnerabilities. In 2016, a security researcher spoke at the Defcon conference about his experiences generating fake QR codes to gain access to airport lounges [27].

Nohl [28] found flight bookings could be accessed using brute force attacks, whereby common surnames and potential passenger name records (PNRs) are repeatedly tried against airline’s online systems. Furthermore, provided with access to a boarding pass, or a photograph of one (which are readily posted on social media sites), the PNR can be read with the use of a barcode scanner (and are printed in plain text on baggage tags). Malicious actors who access booking systems in such a way could change flight dates or destinations, or request refunds, allowing them to travel under the victim’s name. Some airlines also allow name changes. Less obtrusively, they could insert or replace a frequent flyer account number, to harvest the traveller’s points. While the new frequent flyer account must be in the same name as the traveller, some airlines allow name changes on these accounts. Furthermore, the PNR includes the passenger’s name and email address, which may be used to send targeted phishing emails requesting confirmation of frequent flyer credentials or payment details. Nohl was also concerned that GDS’ do not properly authenticate users accessing PNRs, do not rate limit attempts to access the system, therefore allowing the brute force attacks to occur, and do not log when PNRs have been accessed, making unauthorised access harder to detect. Nohl’s work is a proof of concept, showing such attacks are possible. It is unknown if these attacks, or variations of them, had already occurred, although some of the vulnerabilities were reported many years earlier [29].

The aim of this research is to understand the trade in, and use of, fraudulently obtained airline tickets, so as to identify intervention opportunities. As a truly international crime, law enforcement agencies face considerable challenges in investigating and prosecuting these offences, therefore early detection and intervention is particularly important. Furthermore, very little is known about the economy surrounding this market, so identifying how the tickets are obtained will better inform the law enforcement, banking sector, and travel industry response. There is a serious concern that if people can evade existing controls, this crime type might become significantly more prevalent.

## Crime script analysis

Crime scripts outline the significant steps and actions that are carried out in order to prepare for, undertake, and complete a crime. Crime scripts borrow from the concept of ‘schemata’; which are knowledge structures that allow individuals to organise their thoughts or understanding of events or social interactions [30]. Crime script analysis is a method of understanding crime types, and is particularly useful for identifying intervention points where situational crime prevention may be applied.

Crime scripts outline typical behaviours that occur prior to, during, and after a crime event and the actors involved in the process. The universal script involves standardised script scenes or functions arranged in order, namely preparation, entry, pre-condition, instrumental pre-condition, instrumental initiation, instrumental actualisation, doing, post-condition and exit scenes [31]. Crime scripts are not prescriptive, with actors improvising when necessary to ensure their perception of success [31]. These adaptations lead to innovation in relation to offending [32]. Crime script analysis has been applied to a variety of crime types, such as child sex trafficking [33], robbery, auto theft and graffiti [31], drug manufacturing [32], credit card fraud [34], and the illicit trade in pharmaceutical drugs [35, 36]. The method has also been used to look at specific crime ‘actors’, namely organised crime [37] and sex offenders [38, 39].

Hutchings and Holt [6] used crime script analysis to demonstrate how online stolen data markets operate, and the actors involved. The marketplaces involved in that analysis sell stolen data, provide drop,^3^ cashier and money laundering services, as well as other products and services facilitating the stolen data trade, such as malware or skimming devices. A number of actors were identified in this script, including sellers, buyers, suppliers, moderators (who maintain order on the forum and apply the rules), administrators (who organise the platforms and the hosting of the actual forums), and teachers (who write tutorials and provide advice). These roles are not mutually exclusive, as one individual may take on multiple roles, such as seller and buyer, or administrator and moderator. Aspects of travel fraud link back to these blackmarkets, as some tickets are traded here, as well as stolen credit card credentials or compromised account details.

Crime script analysis is particularly useful for complex crimes involving the intersection of different crime types (for example, [36]). According to Hancock and Laycock [37], the value of crime script analysis is that it allows for a better understanding of crime problems. This understanding extends to the types of equipment, the crime facilitators, the locations where various events take place, and the tools and infrastructure required. This knowledge can then be used to inform context-specific prevention and disruption activities. For example, Morselli and Roy [41] used crime script analysis to examine stolen-vehicle exportation operations. They identified key brokers whose removal would result in a significant disruption to the underground market. Similarly, ‘structural chokepoints’ [36] are potential targets for intervention. Structural chokepoints are the components of a criminal operation that are limited in number, yet critical for profitability.

Crime scripts can use various levels of analysis, allowing it to be adaptive to particular crime types. Of particular importance to this research is the ability to focus on particular ‘crime-commission tracks’ ([30], p. 168). Tracks refer to variations in a script based on salient characteristics. This is important because there is more than one way in which travel fraud is carried out. Combining this knowledge with structural chokepoints, it becomes evident that some countermeasures may only address a limited selection of such frauds, while others may have wider applicability. Furthermore, some countermeasures, such as identifying and stopping those travelling, do not address the actors that are obtaining and/or selling the tickets fraudulently.

## Research design and methods

### Scope

The scope of this project has been limited to airline tickets obtained fraudulently from the travel industry. Therefore, relatively straightforward travel fraud occurring in the open market, whereby the vendor receives payment but the buyer receives nothing in return, is excluded. The sole exception to this exclusion is in trying to determine whether sellers operating on online blackmarkets are ‘rippers’, in that they offer the products for sale but do not deliver. It is believed these attempted purchases are less likely to be reported by victims to the police given the shady nature of the marketplace. Also excluded from the definition of travel fraud is ‘friendly fraud’, where a customer claims a genuine transaction is fraudulent. It is further acknowledged that other travel industry segments experience similar fraudulent transactions, such as hotels and car hire. These are occasionally referred to within the context of this research, but are not the primary focus.

### Research questions

This research aims to set out the crime scripts involved in the trade of fraudulently obtained tickets. In doing so, it seeks to address the following questions:

1. What are the methods by which tickets are fraudulently obtained?
2. Are those who purchase fraudulently obtained tickets travelling successfully?
3. Are those who travel on fraudulently obtained tickets aware of the illegality involved?
4. Of those detected travelling on fraudulently obtained tickets, what was the outcome? Were they arrested, charged, and prosecuted? If not, why not?
5. Are those who travel on fraudulently obtained tickets involved in other types of crime?

This research also considers how travel fraud is currently detected and prevented, and other ways this may be approached.

### Methods

As the problem is being addressed from multiple angles (the aspects of the trade, as well as those who travel on tickets obtained fraudulently), multiple data sources are used. These include interviews with the various stakeholders that identify, respond to, or are targets for travel fraud; and excerpts from an online blackmarket where fraudulently obtained tickets are purchased. In addition to these formal methods, the researcher met with a number of law enforcement agents in an informal basis, made detailed notes, and consulted other research. Some information obtained during this research could be used by offenders to improve their methods, or to help them avoid detection. While interesting, such information has not been included in this paper as it is considered prejudicial.

#### Interviews

Interviews were completed with participants who are familiar with various aspects of travel fraud. These include law enforcement, analysts, and industry partners (banks, airlines and industry bodies) who actively detect and investigate various parts of this trade. Particular care was taken to include participants who were familiar with different aspects of travel fraud, such as the booking of the tickets, the interception of travellers, and those familiar with the online trade of fraudulently obtained tickets. In total, 13 interviews were completed, with four participants from airlines, two participants held analyst roles, three were law enforcement agents, two were from industry bodies, one was from a financial institution, and a final participant provided insights from the hotel industry. Many of the participants were recruited through one of the analysts, who is familiar with the landscape, and has the relevant contacts. Further recruitment took place using a snowball sample, in that participants identified additional organisations and individuals to speak to, and through other law enforcement contacts. Interviews took place face-to-face, as well as by telephone and VoIP. Participants were recruited from the US, Asia, and Europe.

The interviews were qualitative and semi-structured. An interview schedule was used, which outlined the topics to be canvassed, however each interview was tailored to the participant, depending on the aspects of the trade they were exposed to. Questions explored the sale of, and travel on, fraudulently obtained tickets, and potential countermeasures.

Ethical clearance was granted for this research, and participants were given an information sheet providing an overview of the research before providing consent. The interviews took between 24 min and 101 min, with an average time of 66 min. All interviews were transcribed, excluding any information identifying the participant or third parties.

#### Online blackmarket

A database of advertisements, buyer feedback, and commentary on a large online blackmarket, which was identified as being the most utilised for trading in fraudulently obtained tickets, was searched. The search terms used are combinations from List A and List B detailed below, as well as the standalone search terms in List C. List A includes travel-related terms, however on their own these terms returned many listings not relevant to this project. List B allowed the results to be more specific to the travel fraud industry, and were designed to specifically elicit the feedback left by unsatisfied customers, who may have been intercepted by law enforcement, or were unsuccessful in their travel attempts. These comments are more forthright, and contain useful information for identifying the methods used. List C includes a number of highly specific terms unlikely to be used out of context relating to the travel industry. The first three terms are names of specific GDS’, which are used by travel agencies for booking tickets. The Airlines Reporting Corporation (ARC) and International Air Transport Association (IATA) are industry bodies for the travel sector.

**Table:** 

**List A** travel airline boarding pass flight hotel expedia	**List B** police europol ripper scam law enforcement cops scammed arrested broker	**List C** Amadeus Sabre Travelport GDS global distribution system Airlines Reporting Corporation ARC International Air Transport Association IATA

Not all search terms or combinations returned matches; however 47 unique threads were identified, covering periods from December 2014 to August 2016.

### Analysis

The interview transcripts, blackmarket content, and the researcher’s notes were analysed qualitatively. Coding of the data was ‘data-driven’ [42], broadly based on the universal script scenes, tracks, and actors as put forward by Cornish [31]. NVivo, a qualitative data analysis program, was used to classify and sort the data. The results section outlines actors and script actions relating to the travel fraud process, as identified in the data. Quotations from participants and the blackmarket are provided verbatim.

## Scripting the travel fraud process

There is no single script when it comes to travel fraud, but rather different tracks that overlap and intersect with each other. To simplify the complexity involved in this trade, this crime scripting exercise divides the ‘universal script’ into three ‘Acts’. The first Act relates to ‘preparing to travel’, and encompasses the preparation, entry, and pre-condition scenes. The second Act, ‘the middle-men and their methods’, includes the scenes relating to instrumental pre-condition, instrumental initiation, instrumental actualisation, and doing. The final Act, ‘(attempting to) travel’, includes the post-condition and exit scenes. Each Act consists of different tracks and actors, which provide options for how the script may develop (like a ‘choose your own adventure’ book).

### ACT 1: Preparing to travel

The main objective in preparing to travel is to purchase a flight ticket from a travel service provider.

#### Actors: Victim traveller; complicit traveller; mule handler; human smuggler/trafficker; re-seller

There are five main actors identified as preparing for travel, two are actual travellers, and three are preparing others’ travel. The two travellers are distinguished by either being a **victim traveller**, who is unaware the ticket has been obtained fraudulently, and a **complicit traveller**, who is aware. One participant estimates victim travellers account for five to 10% of those travelling on fraudulently obtained tickets, although, as will be discussed, this can be hard to verify:

From the perspective of people that are purchasing them, and this is our finding based on the actions so far, between 90 and 95% of the travellers were fully aware, or at least should be aware that it’s criminal activity. Between 10, but we think it’s more close to 5% of the people, were really cheated, either online travel agency or the criminal service was organised so well they were not able [...] to recognise that it’s actually criminal activity behind (participant LE1).

The third and fourth actors are the **mule handler** and **human smuggler/trafficker**, who are organising travel on behalf of others. The actors travelling on these tickets, the mules, smuggled and trafficked, will enter the script in Act 3. Human smugglers facilitate the movement of people from one place to another, however those travelling are doing so voluntarily (e.g. illegal immigration or seeking asylum). On the other hand, human traffickers coerce, threaten, force, or deceive those they are transporting (e.g. sexual or labour exploitation). The fifth actor is the **re-seller**, who purchases the tickets and sells them to others, most likely other victim travellers, mule handlers or human smugglers/traffickers.

#### Track: Online blackmarkets

There was evidence that complicit travellers, mule handlers, and re-sellers use online blackmarkets. There was no evidence that human smugglers/traffickers are buying tickets here, although the possibility remains. These actors are, or should be, aware the tickets available on these blackmarkets are obtained fraudulently. Advertisements for travel services, along with feedback from others who have done, or attempted to do, business with them are listed. Buyers liaise with the seller, who will typically ask them to provide them with a screenshot (an image file) of the booking they require, as well as the details of those travelling. An example advertisement is:

Greetings to the community. We are a team of profesionals at the travel industry who have been in business for over 2 years with resellers all over the globe. Now we want to expand our business offering our worldwide travel services for flight and hotel bookings to the […] community. Offering the opportunity to its members to enjoy life to the fullest and make their dreams come true using our services to get in a travel adventure to a exotic beach a weekend on a 5star resort a getaway with your beloved ones. Be my guest to try our services to get the best out of a trip in a healhty safe and affordable way […] Live the life of a Highroller that you deserve enjoy life to the fullest and travel the world with your beloved ones sharing memories that will last for a lifetime! Benefits of booking with us. Great and affordable experiences with worldwide flights and hotels at 25% of retail valie. Our customers safety and satisfaction is our #1 priority! Friendly customer support active on ICQ/Jabber to answer any questions. No legal risks involved OUR SERVICES ARE NOT CARDED! To arrange our bookings in a safe and long lasting manner we exchange various gift cards and vouchers from several companies and airlines to provide a safe and satisfactory services at a great price! HOW TO BOOK WITH US. Booking request must be done with 72 h in advance. First capture screenshot of the trip you want from any agency […] Upload it on image upload site(anony.wsanonfiles.com) Request us a custom listing so that we will create a custom listing for the specific amount for you. Then provide passenger details as follows. First Name- Donald Last Name- Trump Dob-1/Nov/1955 As soon as the order is done we will provide you booking details for lfights and confirmation voucher for hotels. Thats it just confirm it with airline website or calling hotel and you are ready to go […] (blackmarket post).

Payment is made directly to the seller, or through the escrow service offered by the blackmarket. The escrow service receives the payment from the buyer, and transfers it to the seller once they receive notification they are happy with the transaction. Payment is usually made using alternative currencies such as Bitcoin.

#### Track: Fake travel agency

Fake travel agencies trick unsuspecting ticket buyers into becoming victim travellers. Fake travel agencies have advertised online through paid advertisements on social network sites and search engines; websites returned through searches; through newspapers and classified advertisements; on the radio; and through posters displayed at airports. One participant explained how some of the fake travel agencies operate:

Many of the airlines, including ourselves, have experiences where these companies are paying for ad space on Google so that when you do a search for [Airline], their paid ad comes up before our website comes up. And so, their phone numbers are the first thing that people see, so the customer calls those phone numbers, but they are not us. They are not booking with us (participant AIR1).

In this example, the agency pays for Google AdWords, so their advertisement appears above that of the legitimate airline in search queries. The victim traveller is told they are dealing with the actual airline, and books the ticket as they would normally expect to, providing their personal information, flight information, and payment details.

#### Track: Insider

Insiders work in legitimate travel agencies and airlines. Insiders can take on a variety of roles, including employees, contractors, or third party suppliers, who misuse their privileges. While this track seems similar to the *fake travel agency track*, it differs in that an employee of a legitimate provider carries out the fraud, for example:

We do get rogue employees, where they’re, on the side, taking in cash from customers and then inserting compromised cards to pay for the ticket. That is definitely always a concern (participant IN1).

#### Track: Word-of-mouth

All five actors, victim travellers, complicit travellers, mule handlers, human smugglers/ traffickers and re-sellers may also obtain their tickets through word-of-mouth. Introductions are through close-knit communities, informal networks, and even through church groups:

But there are also tickets sold in communities, for example, some passengers who have been interviewed say that they get the tickets through their church (participant AIR4).

Often there is an element of trust, in that the seller has been recommended through others. In some instances, they are friends and family members. On the other hand, some travellers purchase tickets from people they have only just met. In relation to re-sellers and mule handlers, there was evidence in the blackmarket that trades were also taking place off-forum, with those who had developed business relationships with the sellers.

### ACT 2: The middle-men and their methods

The first Act explores the different options by which tickets are purchased. In Act 2, the methods by which these tickets are obtained are outlined.

#### Actors: Blackmarket seller; fake travel agent; rogue employee

In many cases, the person travelling on the ticket is not the person who actually obtained it from the affected airline or travel agent. In Act 2, a number of new actors are encountered: the ‘middle-men’. These include the **blackmarket seller**, the **fake travel agent**, and the **rogue employee**. The reader may recognise that some of these actors correspond to the tracks outlined in Act 1. For instance, the blackmarket seller operates within the *online blackmarket track*, the fake travel agent within the *fake travel agency track*, and the rogue employee is the actor the *insider track*. This Act will demonstrate the methods used by these actors, which again may overlap and intersect across the different tracks.

#### Track: Compromised credit cards

Compromised credit cards are used by a number of actors, including blackmarket sellers, fake travel agents, and rogue employees. Compromised credit cards can be used to book tickets directly with the airline, however chatter on the blackmarket indicated that airlines are more likely to detect this type of fraud. Therefore, many blackmarket sellers will instead book with smaller travel agencies without sophisticated fraud detection systems. There are also different places compromised credit cards are used, such as through a booking website, a call centre, in-person at a travel agency, or at the airline’s desk in an airport.

The compromised card data can be purchased from the same, or similar, blackmarkets where the travel services are offered for sale. It is believed many of the middle-men are specialists, and are purchasing the data, rather than obtaining them themselves. This is not always the case, with a police investigation revealing one group obtaining tickets using credentials they had phished themselves.

The *compromised credit card track* is one of the most versatile. It is also one of the more obvious means by which tickets are fraudulently obtained, and therefore the most likely to be detected and cancelled. However, there was evidence of diversification. A number of methods were identified that enabled compromised cards to be used in a way to slow detection and circumvent fraud detection systems. Middle-men have also displaced to new methods, such as those explored in the following tracks.

#### Track: Loyalty point fraud

Loyalty points, also referred to as reward points, frequent flyer points, or miles, are offered by airlines and banks to reward their clients for repeat custom. The loyalty programs have differing rules limiting the movement of points from one account to another, however many can be used to book a ticket in a name other than the account holder, and if the loyalty program is part of an alliance, can be used to obtain tickets with a number of airlines, therefore making detection more difficult.

Participants explained when a loyalty point account was compromised the first thing they see is a change to the email address associated with the account. The credentials to access the account may also be changed, and sometimes the address and phone number stored on the account. After this, the points accumulated in the account are used. It is believed the majority of accounts are compromised because the account holder has used an email and password combination shared with a breached account. Airlines and financial institutions can see the automated testing of multiple username and password combinations attempting to access their systems. It appears these login attempts come from account checkers, which test compromised data against a variety of different online accounts in order to gain access. Social engineering attempts, through social network sites, have also been seen, although it is not believed this works at scale. Some accounts are also compromised by telephone, by calling the bank or airline and impersonating the account holder.

#### Track: Unauthorised access to global distribution systems

Travel agencies book tickets using a GDS to link with the airlines’ computer reservation systems. A number of different companies provide GDS’, including Amadeus, Sabre and Travelport. For a six-year period, a group in the Ivory Coast was compromising these systems, and issuing tickets fraudulently. Phishing emails were sent to travel agencies to obtain their login credentials to the system. As it appeared the affected travel agency had issued the tickets, they would be invoiced for the travel. The travellers were also mainly from the Ivory Coast, and in some cases waited at the airport for the ticket to be set up. While this group apparently stopped in 2014, the use of this method may be ongoing. The method is consistent with other instances of detected fraud, and terms related to GDS’ turned up occasionally on the blackmarket, for example:

- User 1: Soon we will have hacked amadeus logins but we will list this when ready [...]
- User 2: Care you elaborate how are you going to use them? Thank you [...]
- User 1: Book tickets (blackmarket post).

#### Track: Compromised business accounts

Much legitimate travel is for business purposes, and many businesses have corporate accounts with travel agencies for their bookings. Furthermore, some travel agencies only deal with business clients, and some large organisations will have their own internal travel agency. Business accounts can be compromised through a variety of ways, either aimed towards the corporation, or the travel agent. One scheme involves what is known as business email compromise. This either involves the compromise of a legitimate email account, or the creation of an email account that appears to belong to target, such as registering a similar domain name. This email account can then be used to converse with the travel agency, and trick them into issuing a ticket that is then invoiced to the corporate account. Depending on how long it takes to identify, this type of fraud can perpetuate for some time. Corporate booking tools, which allow businesses to make bookings directly through their travel agency’s system, are also vulnerable to attack. Once compromised, they are used to purchase as many tickets as possible before being detected.

#### Track: Identity fraud

There was some speculation on the blackmarket that one method sellers can use is identity fraud. This involves opening a credit account in a fake or victim’s name, and using this to purchase tickets. The advantage of such an approach over the *compromised credit card track* is there is no chargeback when the victim finds unauthorised transactions on their account. However, the downside is this method requires a level of information that is hard to obtain and use in such a way to obtain a high credit limit, and newly created cards used for suspicious transactions such as flights are usually blocked quickly. Therefore, participants confirmed this method is identified occasionally, but not as frequently as compromised credit cards and loyalty point fraud.

#### Track: Voucher fraud

Vouchers include gift vouchers for travel services, as well as vouchers issued by airlines to compensate travellers following a disruption to their journey, such as a delayed or oversold flight. Like loyalty points, vouchers are an internal currency, and are therefore vulnerable to fraud. Rogue airline employees have been found issuing vouchers when no negative experience has been incurred, and not providing the voucher to the intended recipient.

A blackmarket seller was also offering the sale of travel gift vouchers. They claimed to have gift card numbers that had been issued years ago but not used. This vendor attracted a lot of attention, with one of the selling points being the buyer could make the booking themselves, without providing their personal information. However, this seller was only operational for six days before buyers started reporting bookings being cancelled. There are a number of ways disused gift cards might be obtained, including having a rogue employee in the company, or accessing their computer systems remotely. Alternatively, the seller may have lied about their method, and purchased gift cards using compromised credit cards.

### ACT 3: (attempting to) travel

#### Actors: Victim traveller; complicit traveller; mule; the trafficked/ smuggled

The victim traveller and complicit traveller reappear in Act 3, along with new actors, the **mule** and the **trafficked/smuggled**. The victim traveller may be journeying for a variety of reasons, just like any other traveller. Similarly, the complicit traveller may be travelling for personal reasons, such as taking a holiday or attending a family emergency. One potential complicit traveller identified in a blackmarket post they were planning their honeymoon. However, some complicit travellers carry out other criminal activities. Mules may be aware they are facilitating crime, or they may be victims, such as ‘romantic mules’, who believe they are in a relationship with their handler. Unlike complicit travellers, it appears mules and the trafficked/smuggled are provided with the tickets, rather than obtain them themselves, and may not be aware of their fraudulent origin. It was believed the majority of the smuggled are trying to escape hardship, sometimes trying to reunite with their families, whereas many of the trafficked are facing sexual exploitation.

The tracks present in Act 3 provide potential experiences the traveller may encounter, including *cancelled bookings*, being *detained by law enforcement*, *providing identification*, and *travelling successfully*. Act 3 also contains a track for *involvement in other crime*.

#### Track: Cancelled booking

The most common way a journey goes awry is if fraud is detected and the ticket is cancelled. This can happen shortly after booking, or shortly before the flight (although sometimes these may be the same time). The latter appears to have the most impact, as it does not allow time to seek alternative arrangements. Another outcome of cancellations is that complicit travellers and mule handlers complain using the feedback mechanism on the blackmarket. If a blackmarket seller is experiencing many unsuccessful bookings, they will be labelled a ‘ripper’ or ‘scammer’, and in some cases will be banned from the marketplace.

Airlines are juggling two sometimes competing imperatives, one being fraud detection and prevention, while the other is the customer experience. One concern airlines have when it comes to cancelling flights is the possibility of false positives, whereby a ticket that appears to be fraudulent is in fact legitimate. An alternative to cancelling a ticket, particularly if the airline has not been able to formally confirm a transaction is indeed unauthorised, is to put the transaction on hold, and request an alternative form of payment at the airport prior to boarding. This method also allows the airline to gain some intelligence about how the ticket was obtained. As the original payment is then reversed or not processed, legitimate travellers are only paying once, and victimised card or account holders do not suffer any loss. However, those who have purchased fraudulently obtained tickets will essentially pay twice for their journey, if they wish to board the flight. Airlines try to minimise the impact on victim travellers, but they can be hard to differentiate from complicit travellers. Advice on the blackmarket for travellers who face this scenario is ‘you pay no argument/questions asked’.

#### Track: Detained by law enforcement

In some cases, law enforcement become involved, detaining and questioning those travelling on fraudulently obtained tickets. This may be part of the Global Airline Action days, run with Europol and other industry and law enforcement partners, part of a focused investigation, or done on an ad hoc basis. As identifying who is truly a victim traveller is problematic, when a traveller is detained, they are rarely charged with any crime. These difficulties have not gone unnoticed on the blackmarket. While they have shared media releases relating to the global action days, they also note law enforcement are rarely involved, and when they are, the traveller, not the person who obtained the ticket, is targeted:

Although it looks like theyve arrested purchasers of said service but not the guys running the business in the first place... (blackmarket post).

However, participants indicated that law enforcement are starting to take more notice of travel fraud, and even when a passenger was detained with no charge, this ultimately causes some disruption to the person who had organised the ticket.

Differentiating complicit travellers and victim travellers is complicated as the former can easily claim to be the latter. The blackmarket contain advice about how to do this if detained, including preparing fake correspondence before travelling. The provision of fake correspondence and receipts has even been advertised as a service on the blackmarket.

#### Track: Providing identification

The use of fake or stolen identification was discussed on the blackmarket, and the consensus was, for flights at least, the traveller’s genuine passport should be used. This may seem counterintuitive at first, particularly as fake identification can be obtained in these blackmarkets, and their use hides the traveller’s real identity if the fraudulent ticket is discovered. The reason fraudulent or stolen identification is not recommended is, as identified in the *detained by law enforcement track*, travellers can easily claim they are victim travellers, and travelling with fake identification would not lend any credence to this assertion. Indeed, travelling on false identification would increase the risk of detection.

Most participants agreed travellers used their own identification, however this is not always the case. It is likely that where a false identification document is used, it would have been used anyway, regardless of how the ticket was obtained, such as to avoid travel watch lists. Some participants hypothesised travellers would be more likely to use a fake name for bookings when travelling domestically or within the European Schengen Area, where identification is not necessarily checked for border control purposes.

However, just because genuine documents are provided, it does not mean all the information provided to the airlines when the tickets are purchased is correct. Advice given on the blackmarket is to change the details slightly, and airlines also advised this happens. It seems that when a passport is swiped at check-in, at least for some airlines, any incorrect information, such as a wrong date of birth, is automatically updated.

#### Track: Travelling successfully

If the ticket is not cancelled, the passenger is not detained by law enforcement, and there are no other reasons the travel did not occur (such as missing the flight) then it is likely the traveller reaches their destination successfully. Complicit travellers who purchased their ticket through a blackmarket will sometimes provide feedback, advising if they travelled successfully or not. Some blackmarket sellers provide a free or heavily discounted ticket when they are just starting out, in exchange for ‘vouches’, whereby the buyers will write a review of the service provided. In other cases, feedback is left for other potential customers, particularly where travel has not been successful.

Another step in travelling successfully is releasing payments through the escrow system, if this was used. However, it is apparent many blackmarket sellers are asking for release of escrow after the flight booking has been confirmed, rather than after the journey. This can lead to problems when the ticket is later cancelled. As mentioned, complaints about unsuccessful journeys can lead to allegations the blackmarket seller is a ripper when payment is not refunded.

#### Track: Involvement in other crime

As has been alluded to throughout this script, some of those travelling on fraudulently obtained tickets are known or suspected to be involved in other offences. In addition to human smuggling and trafficking, other crimes travellers are known to be involved in include: theft, including pickpocketing and shoplifting from airport stores; smuggling cash and contraband, such as drugs, cigarettes and tobacco; facilitating money laundering, such as opening bank accounts in other countries; and credit card fraud, including making transactions with compromised cards, and operating skimmers. With some of these additional offences, there are elements of organised crime and co-offending present.

There were suspected links to terrorism financing, however there was little evidence to support this concern. There were also claims the movement of people and proceeds from the sale of some fraudulently obtained tickets were linked to an alleged terrorist group. Some of the offences listed above are more readily ascertained than others, particularly when contraband is involved. On the other hand, mules opening bank accounts, and human smuggling/trafficking may be less likely to be detected. Furthermore, some contraband, such as credit card skimmers, may be difficult to recognise by those who are untrained.

## Current and emerging approaches to detection and investigation

There are a number of approaches currently used to detect, prevent, and investigate travel fraud. The first approach is detecting tickets from being fraudulently booked to begin with, and cancelling those tickets that have been booked to prevent the travel from taking place. This section also explores law enforcement action against travel fraud, particularly outside the Global Airline Action Days, and ways information is being shared within the industry.

### Detecting and cancelling fraudulent bookings

Fraud detection systems are used to detect fraud at the time of booking. These systems use algorithms to score the potential risk of a booking. Risk factors can include details about the person travelling, the way the booking was made, the route being flown, and associated variables, such as time before travelling. A risky booking can then be reviewed, and attempts can be made to confirm its legitimacy, by making enquiries with the card issuer. If there is confirmed fraud, or significant doubt, the booking is cancelled, or another payment method is requested.

Airlines and travel agencies may run fraud detection systems against their bookings. Furthermore, financial institutions attempt to detect fraudulent transactions on their cards. Banks and airlines sometimes use a third party vendor for their loyalty programs, which may also use their own fraud detection systems. Multiple parties operating their own fraud detection systems is beneficial, as while one party, such as an airline, may see deeply into fraud on their own systems, another party, such as a bank or third party vendor, may see more breadth, with attempted fraud across multiple targets.

Another organisation that has the ability to see fraud across a wide number of targets is the Airlines Reporting Corporation (ARC), which plays an active role in detecting fraudulent travel booked with travel agencies. ARC facilitates the payment for tickets between US travel agencies and US and international airlines. As a result, they can see who is booking tickets, with which agency and airline, and what route is being travelled. Due to the volume of data, ARC mainly reviews transactions relating to high-risk locations associated with large amounts of fraud. Outside of the US, IATA has a similar financial collection function to ARC.

Blacklists, or name traps, make up a part of fraud detection systems, to identify names or other identification information associated with fraudulent transactions in the past. The topic of blacklisting generated many comments on the blackmarket, particularly what the outcome was if a name was blacklisted. One person posed a question enquiring if blacklists target the vendor or the passenger. The response indicated that even if a traveller is blacklisted, they are still able to travel if they use legitimate payment methods. Another commentator compared a number of blacklisting practices, claiming they were often easily circumvented or non-existent. One concern with permanently blacklisting travellers is that victim travellers are not aware of the illegitimacy of their tickets. Furthermore, if limited or incorrect information is provided, making an accurate match can be difficult.

Fraud detection systems are not foolproof. In particular, they have mainly been developed to detect fraudulent credit card transactions, so few will detect more elaborate frauds. Furthermore, they are not always run against telephone or in-person bookings, or against changes to bookings. Detecting, and confirming, fraud can take some time, and the criminals have found booking close to departure can be successful. ARC only sees transactions after the end of each day, due to the way they are batched and sent for settlement of payments between the travel agencies and airlines.

One of the main difficulties with identifying fraudulent transactions relates to receiving confirmation from a bank a credit card transaction was not authorised. This can take time, particularly if multiple time zones and languages are involved. This issue is compounded when there is no incentive for the bank to detect fraud themselves, or respond in a timely fashion. If the transaction is fraudulent they suffer no financial loss, with the airline or travel agency generally liable for the chargeback.

Further ways service providers try to prevent tickets from being booked fraudulently include identifying and resetting passwords for compromised loyalty point accounts. One participant advised they see attempts to validate large numbers of credentials over short periods. The level of security for access to loyalty point accounts varies, with some just requiring a short PIN, and others a password. However, at least one airline advised they were moving to two-factor authentication.

Some airlines and travel agencies are now accepting Bitcoin as a payment method. This poses potential new avenues for fraud detection -- not only detecting compromised Bitcoin, but potentially the use of block chain analysis to identify Bitcoins associated with known bad transactions such as money laundering.

### Law enforcement involvement

Getting law enforcement involved in combating travel fraud has been an ongoing process. Europol has taken the lead, coordinating the Global Airline Action Days since 2013. These have gradually expanded, to increase the number of jurisdictions involved. The action days are run in conjunction with local law enforcement, airlines and financial institutions, to detect fraudulently obtained tickets, and those travelling on them. Outside the global action days, it can be challenging to get law enforcement involved. Some of the issues include:

- The low dollar amount for one-off purchases. This has been noted in the blackmarket, where actors reassure each other the risk is low for this reason. Some participants said they could only get law enforcement involved when losses started to reach multiple hundreds of thousands of dollars.
- Fraud is not seen as a priority for law enforcement. Furthermore, adding complex international aspects can make investigations unattractive to pursue.
- A lack of international contacts. Like many interactions in life, having trusted relationships can make communications easier. However, due to the global nature of travel fraud, making connections with law enforcement all around the globe, with different time zones and languages, can prove difficult. While airlines advised they often have good relationships with their local law enforcement agencies, this is more difficult in non-local jurisdictions.
- Some policing agencies do not want to, or cannot, get involved in cases not directly relating to their own country, such as when an international traveller is arriving on an airline from another country, and the affected financial institution is also overseas.
- There can be local jurisdictional issues. For example, in some countries the police officers working in airports have no jurisdiction over fraud matters.
- A lack of awareness about travel fraud. Many law enforcement officers are not versed in complex frauds, and there is little institutional knowledge. Even when law enforcement detains a traveller, they may not know what questions to ask.
- Problems with being able to charge those travelling. This issue is compounded when travellers are, or claim to be, victims who do not know the ticket they are travelling on has been obtained illegally. Furthermore, some law enforcement are unlikely to charge someone with credit card fraud if they are not in physical possession of the card, even for card-not-present transactions.

There is also seldom any action taken after a flight has already occurred. Of course, this can be challenging for law enforcement, because in addition to the points covered above, the location and residence of the traveller can be difficult to identify. Detaining a traveller during their journey is comparatively much simpler, due to their physical presence at the time. Furthermore, for airlines, valuable time and effort would be required, which can be better spent trying to detect and stop travel that has not yet flown. However, work is progressing to identify prolific travellers, who travel many hundreds of times on illegitimate tickets, by both law enforcement, and the travel industry. One of the biggest issues with policing travel fraud may be taking action against those responsible for making the fraudulent bookings, rather than the travellers.

There were indications that efforts to involve law enforcement were improving. Participants credited Europol and the National Cyber-Forensics and Training Alliance for helping to address this issue. Some law enforcement agencies had also started to recognise the problem, with the one cybercrime unit opening a reporting platform to receive incidents from the travel industry.

Of course, the problem is not solely about getting law enforcement involved. A related concern is the travel industry not reporting this type of fraud to the police. Due to the volume of fraud identified, and many bookings being made at the last minute, the most common response is to cancel the ticket, without any reporting. It was also believed the time and resources required to cooperate with a police investigation would be too extensive and expensive to be of value. In some countries, trust in law enforcement may be lacking, hindering cooperation with the travel industry.

### Sharing information

Due to the number of different airlines, travel agencies and financial institutions involved, everyone holds a different piece of the puzzle and it is difficult to see the whole picture. One way the response can become better coordinated, for both fraud detection and enforcement, is to share information, within the bounds of the law relating to personal data. There are concerns about the sharing of personal data, particularly when the jurisdictions and legal frameworks differ. However, there are often exemptions for the prevention of fraud, and passing information to law enforcement. Therefore, it may be possible to share data under cover of a formal contract. In all cases, local legal advice would need to be taken. The benefit of data sharing, and combatting travel fraud as a collective, is becoming recognised. While these organisations may be competitors, the value of the collective information is greater than its individual parts. As offenders may displace from one travel provider to another, learning from others’ experience can mean that fraudulent bookings can be detected straightaway, before chargebacks start being incurred. In recognition of this, sharing networks have started to spring up globally. These include:

- Listservs (email distribution lists) used to share data associated with fraudulent bookings. As they also share information relating to loyalty point compromise, data sharing partners include financial institutions, as well as airlines, law enforcement, and industry bodies.
- The European Airlines Fraud Prevention Group. The group communicates and meets regularly, sharing data to assist in fraud detection. This group was the original group working with Europol with the global action days. As the name implies, the group is made up of European airlines.
- Similar fraud prevention groups based in Asia and Latin America, based on the European group. These fraud prevention groups are organised under IATA.

One of the potential barriers to data sharing is trust. Organisations need to trust the data is only being used for the purposes for which it is being shared. They also need to trust whoever is centrally organising the data sharing platform. Participants advised they had started to develop better trusting working relationships with others as a result of information sharing. Some organisations seemed more comfortable with accessing information, but not sharing it themselves, but developing that trust was seen as a step towards breaking down that barrier. There were hopes for more fraud prevention groups to be set up, and that data would start to be shared across travel providers globally.

As well as sharing information between travel providers, the benefits of sharing information with financial institutions was acknowledged. This was particularly the case when attempted bookings are not processed due to fraud, but the bank is not informed of the attempted transaction. The card may be compromised, but if the issuing bank is not aware, they will not cancel the card or monitor it for other suspicious transactions.

Increasing data sharing with police may also be of value when it comes to triaging reported fraud. Europol has a database of suspects linked to other crimes, such as trafficking, illegal immigration, and financial crime. This database has been of use to link instances of travel fraud to other forms of criminality. Furthermore, the jurisdictional boundaries surrounding policing agencies means each may hold different pieces of information. Local police may hold some data, some may make it into national police databases, and, as identified, some may be shared internationally. Of course, the right questions need to be asked in the first place, and a standard questionnaire for use by law enforcement may be a good place to start. Only data properly recorded, shared, and searched will be of later use.

Because of the way participants were identified to be interviewed for this research, everyone was highly connected and involved in data sharing. However, it was acknowledged there are many involved in the industry that do not yet benefit directly from such an approach.

## Potential alternative intervention and disruption opportunities

Crime script analysis is used to identify the intervention points where crime disruption initiatives may have the best effect. One way to focus on structural chokepoints relating to the online trade of illicit goods is to identify those aimed at disrupting the act, the actor, and the marketplace [43, 44]. In relation to travel fraud, the act is obtaining tickets fraudulently, the actors are those buying, selling and travelling on tickets, and the marketplace is where the fraudulently obtained tickets are sold. There is not scope to identify all the different ways these methods may be detected and prevented within this section, particularly as some are likely to have already been implemented. However, during the interviews it became evident there were some initiatives that may be of use to address particular chokepoints, which were either not used, or were underutilised.

### Interventions aimed at the act

Known methods for obtaining tickets fraudulently include the use of compromised credit cards, loyalty point fraud, unauthorised access to the global distribution systems, compromised business accounts, and to a lesser extent, identity fraud and voucher fraud. In relation to compromised credit cards, one participant advised 20% of airlines did not verify credit cards through the CVV, the three digits located on the back of the card. Requesting the CVV at the time of transaction may be one step that can be taken to improve security. While this is useful, it is not foolproof, as the CVV may also be compromised. In addition, recent research has found bots may be utilised to guess the CVV and expiration date of Visa cards in as little as six seconds [45].

A number of disruption mechanisms may be useful for preventing loyalty point fraud. Some participants noted the first thing they saw occur on accounts once they had been compromised was a change of email address, and sometimes other contact details such as phone number. Therefore, making it more difficult to change the details on the account for someone who is not the account holder could provide better security. Some form of multifactor authentication may be applicable to address this problem. For example, changing the telephone number may require the account holder to confirm the change via an email sent to the address on the account. Conversely, changing the email address may require an authentication code sent to the account holder’s phone to be entered. Changes to the contact details made over the phone, as well as using the online system, would also need to be verified, to minimise displacement. Implementing some kind of multi-factor authentication for access to accounts could also be a future consideration, although the specific needs of travellers do need to be taken into account. For mobile authentication, this includes access to mobile phone networks and the high cost of roaming charges, while for physical tokens there may be limitations on what account holders will want to carry with them.

Some participants also advised they could see credentials, apparently breached elsewhere, being checked against their loyalty account login systems for a match. Automated checkers are also used for compromised credit cards, to determine if a card is still active. Potential countermeasures against credit card checkers may also be of value to prevent the automated checking of credentials. Anti-automation methods suggested by Peacock and Friedman [46] include the use of CAPTCHAs, reputation methods, proof-of-work problems and real-time polymorphic web content. This would require offenders to test credentials manually, thereby increasing the effort, cost and time involved.

There could also be changes to how cancellations are handled to have a greater impact on travellers using fraudulently obtained tickets. It seems the most disruption is caused when flights are cancelled immediately before boarding, as cancelling them beforehand provides time to make alternative travel arrangements. Furthermore, this is likely to have a negative effect on the reputation of blackmarket sellers, who in some cases are requesting escrow funds to be released prior to the flight. One way airlines could change their practice is to only notify cancellations due to fraudulently obtained tickets at the time of check-in. The argument against this is victim travellers would be left stranded. While they may have to pay twice in order to travel, this is likely to be the same outcome if the ticket is cancelled earlier.

It is noted some of these methods, and others, that may disrupt fraudulent bookings require an investment on behalf of the travel provider. For airlines to invest in better security, there is a recognised need for a change in the perception at the corporate board level that fraud detection is not an expense, but a saving.

### Interventions aimed at the actor

Interventions aimed at particular key actors may have a disproportionately large effect. Some participants believed a small number of actors are associated with the majority of fraudulent sales and re-sales. These actors are perhaps the targets law enforcement should be focussing on, rather than only those who are travelling on the tickets they obtain.

For key actors operating on online blackmarkets, where attribution may be difficult, there are other ways their businesses may be disrupted. These methods are aimed at creating the appearance of mistrust between buyers and sellers, referred to as ‘lemonising the market’. A lemon market is one in which there is quality uncertainty; therefore, those selling quality products are unable to differentiate from sellers with poor-quality products and cannot compete with their low prices [47]. As a result, engaging in the market would increase the effort and cost of crime for buyers and reduce their expected benefits. Markets may be lemonised using Sybil and slander attacks, leaving comments highlighting the dangers of travelling on fraudulently obtained tickets, and providing false information. In relation to travel fraud, sellers are usually provided with information about who is travelling. Sowing mistrust within the blackmarket may have people thinking twice before providing personal information. However, it is important to consider legitimacy and due process when undertaking such disruption activities [44].

There was some indication on the blackmarket that actors are aware blacklists are not permanent. Those communicating this indicated it was important to them. They wanted to make sure that if they were detected, they would still be able to fly with that airline in the future. Therefore, airlines may want to consider a policy that travellers repeatedly detected travelling, or attempting to travel, on fraudulently purchased tickets be permanently blacklisted, regardless of whether the later ticket is purchased illegally or not. While this may mean airlines are missing out on a small amount of future revenue, it also increases the cost to potential complicit travellers.

It is apparent that employee misuse and corruption are elements enabling some forms of travel fraud. The level of employee misuse was not known, and it is likely to be under-detected. Furthermore, there is some opportunity for law enforcement corruption, which can be particularly hard to detect. This can included systematic corruption, such as providing tips about when not to travel, or can be opportunistic, such as accepting a bribe. It has been argued that successfully addressing corruption requires a systematic approach by governments [48]. Broad law enforcement anti-corruption initiatives, that are aimed at the issue overall rather than on specific crime types, include establishing an anti-corruption agency, providing adequate pay, staff rotation, ensuring staff are not politically appointed, creating disincentives for corruption, removing opportunity, increasing transparency and addressing cultural issues within the organisation [48]. In relation to insiders, restricting the ability to issue tickets or internal currencies, having systems in place to detect misuse, and the use of background checks at the time of recruitment may also be beneficial.

One participant suggested a way to prevent people buying tickets fraudulently was to reduce the demand for cheap tickets. Along this line of thinking, there is a need to reduce global inequalities and the mistreatment of people, such as the labour and sexual exploitation that is associated with human smuggling. Similarly, legitimate ways for people to seek refuge, rather than travelling illegally to seek asylum, could reduce demand. Of course, such approaches require political will, which seems to be in short supply.

### Interventions aimed at the marketplace

The marketplace for fraudulently obtained tickets includes the online blackmarket where sellers advertise, as well as the fake travel agencies, and communities where tickets are sold by word of mouth. One way to disrupt fake travel agencies is website takedown. Websites can be taken down by requesting registrars suspend the domain name, or hosting provider remove the offending site [49]. Ownership of website takedown can be problematic when there is no specific brand involved. For example, a website imitating a legitimate airline or travel agent may come to the attention of, and by dealt with by, the brand owner. However, fake travel agencies that do not imitate any one particular brand are perhaps less likely to be requested to be taken down, even though they cause harm to the industry overall [50]. IATA and ARC accredit travel agencies, and could potentially take a role in requesting websites be taken down that have been linked to fraudulent tickets and are not accredited with them, or pay for commercial takedown companies to do this for them. IATA and ARC may also be in a position to provide advice to the travel industry about how to request websites that are copying legitimate travel businesses to be taken down.

It can be difficult for potential travellers to verify if an online travel agency is legitimate or not. As stated, travel agencies are accredited through IATA and/or ARC. However, knowing to check for accreditation, or being able to identify where accreditation is faked, is unlikely for the average consumer. Even fake travel agencies can have websites that appear professional, and some participants noted the fake travel agencies were purchasing Google AdWords in order to promote their websites.

Here, Google has some past experience in how to disrupt the trade in illicit goods. Google bars unlicensed pharmacies from purchasing AdWords, by cross-referencing against a list of law-abiding pharmacies. Google does this to comply with US legislation relating to the supply of prescription drugs. Therefore, the organisation has the capability, but not necessarily the incentive, to ensure advertisements for travel services come from organisations accredited with IATA and ARC. It is possible the collective power of the travel industry will carry some clout with Google to implement such a preventative measure. While Google’s intervention did not completely stop the illicit trade in pharmaceuticals, their actions did mean operators had to find alternative ways in which to drive traffic to their online stores [36].

## Conclusion and discussion

Methods by which airline tickets are fraudulently obtained are usually compromised credit card details and loyalty point accounts, gaining access to the global distribution systems by compromising travel agencies, and to business accounts by compromising organisations. Identity fraud may play a role, however this is more difficult to carry out successfully, and involves a greater amount of effort than other methods. The fraudulent trade in travel gift vouchers has also been identified as a means for obtaining illicit airline tickets.

While some fraudulently obtained tickets are detected and cancelled before they are flown, it is apparent that many travellers using these tickets travel successfully. In some cases, the booking is not detected in time, and sometimes it is not known to be fraudulent until some time later, when a chargeback is received. Those obtaining tickets use a variety of methods minimise the likelihood the booking will be detected before the flight takes place.

Those travelling on fraudulently obtained tickets purchase them through online blackmarkets, fake travel agencies, by word of mouth, or through genuine travel agencies where an employee is passing through stolen credentials. In some cases, they have not purchased the ticket directly themselves, but are provided the ticket by a mule handler, smuggler or trafficker, or by co-offenders they are committing other crimes with.

When a traveller is detected travelling on fraudulently obtained tickets, they invariably claim they are unaware of the ticket’s origin. They claim the ticket was obtained through a family member of friend, or a travel agency that must have been dodgy. While some of the claims of innocence are likely to be true, buyers on the blackmarkets are being coached to make similar assertions, and there are suggestions they falsify receipts and communications to back up their story. This causes problems for law enforcement when it comes to taking legal action against travellers, and subsequently there are few prosecutions.

Travel fraud has been linked to a number of other crime types. These include smuggling contraband, including drugs, cash, and cigarettes and tobacco. In some cases the movement of people has been linked to human trafficking, including for sexual exploitation, human smuggling, and illegal immigration. Property crimes include theft and robberies, including within airports, such as pickpocketing and organised shoplifting. In addition to smuggling cash, people can be transported to a country for the purpose of opening bank accounts, which are used for money laundering. And finally, further credit card fraud can be enabled through travel fraud, such as setting up skimmers and travelling to new destinations in order to use compromised card data.

Travel fraud has changed over time. This change has been driven by new opportunities, as well as displacement, as old methods have become more difficult to carry out successfully. This explains why there are so many different methods for fraudulently obtaining tickets. It is likely offenders will continue to innovate, taking advantage of different ways the travel industry serves their legitimate customers, as well as new, and existing, fraud methods that arise.

Airlines and the travel industry attempt to detect fraudulent bookings before travel has taken place. One way of doing this is the use of fraud detection systems, which are often paid-for services offered by the financial sector. There may be ways to improve the scope for what is monitored for fraud detection, such as by expanding the information sharing groups that have been set up. These groups have been built upon established relationships, and the sharing of information is a sensitive issue, particularly with competitors. However, to quote Aristotle, ‘the whole is greater than the sum of its parts’. Organisations may see the benefits of quickly detecting travel fraud outweigh the cost of letting competitors know details of when they have been defrauded. Furthermore, the sharing of information between the travel and financial industries can be improved. This includes banks providing timely confirmation if a card has been compromised, and conversely, being informed if a fraudulent purchase has been attempted, but voided before the transaction has been completed.

Some of the challenges for law enforcement relate to understanding the travel fraud problem. The use of proforma interview schedules, to ask detailed and pertinent questions and seek evidence, may be beneficial. Feeding the intelligence gained back to an international system would also be advantageous, so it can be analysed strategically. Ultimately, focusing on those obtaining tickets, rather than travellers, may have more impact. Some law enforcement are also providing central points of contact for receiving reports of fraud from the travel industry.

Some of the issues when it comes to detecting and preventing travel fraud come down to incentives for the various players. For example, participants perceived financial institutions as having no incentive to detect fraud, or to quickly confirm fraudulent transactions, as the cost is borne by the merchant. Law enforcement may be reluctant to pursue travel fraud cases due to their transnational nature, which makes it difficult to obtain evidence and prolongs the investigation. Furthermore, disrupting the advertising of fake travel agencies means the advertising platform is missing out on revenue, and increasing their costs. These problems may be addressed by asking how organisations might be rewarded for being more proactive, or conversely, what they might suffer as a consequence of not taking action when it is in their power to do so.

Finally, as Nohl’s [28] work demonstrates, travel is booked using legacy systems. Even old systems pose new threats, and travel fraud is likely to continue to evolve in new ways. The challenge will be in identifying new methods and being able to respond appropriately, and timely, fixing vulnerabilities in these systems as they arise, or detecting them before they are exploited.

This research has attempted to overcome the significant difficulties associated with this challenging area of research. However, a number of limitations in the research design are identified. First, while participants were from the US, Asia, and Europe, there may be regional differences in the characteristics of the frauds that have not been identified. Second, this analysis only looks at the current state of play. It is likely that travel fraud will change over time, with new opportunities and in response to crime prevention and fraud detection methods. Furthermore, it is possible additional methods to commit travel fraud are more successful, and have not been identified. And finally, as this research is exploratory, it is not attempted to quantify the various aspects that have been identified.

## Footnotes

1 Anonymity networks are networks of encrypted connections, with random pathways through relays, designed so observers cannot tell where data are coming from or where they are going. The onion router, or Tor, is one such anonymity network [7]. Tor includes ‘hidden services’, which do not reveal the physical location where a website is hosted. Another anonymity network is I2P, with hidden service equivalents called ‘eepsites’ [8].

2 ‘Rippers’ are dishonest traders who cheat buyers in online blackmarkets [11].

3 ‘Drop services’ receive fraudulently purchased goods, and can exchange them for cash [40].
